# Beta-frequency electrophysiological bursts: BOLD correlates and relationships with psychotic illness

**DOI:** 10.1192/bjo.2021.151

**Published:** 2021-06-18

**Authors:** Paul M Briley, Elizabeth B Liddle, Karen J Mullinger, Molly Simmonite, Lena Palaniyappan, Richard W Bowtell, Tom White, Marije Jansen, Vijender Balain

**Affiliations:** 1 Centre for Translational Neuroimaging in Mental Health, Institute of Mental Health, School of Medicine, University of Nottingham, Nottinghamshire Healthcare NHS Trust; 2 Centre for Translational Neuroimaging in Mental Health, Institute of Mental Health, School of Medicine, University of Nottingham; 3 Sir Peter Mansfield Imaging Centre, School of Physics and Astronomy, University of Nottingham; 4 Department of Psychology, University of Michigan; 5 Department of Psychiatry, University of Western Ontario, Robarts Research Institute, University of Western Ontario, Lawson Health Research Institute; 6 Sir Peter Mansfield Imaging Centre, School of Physics and Astronomy, University of Nottingham; Peter Liddle, Centre for Translational Neuroimaging in Mental Health, Institute of Mental Health, School of Medicine, University of Nottingham; 7 Centre for Translational Neuroimaging in Mental Health, Institute of Mental Health, School of Medicine, University of Nottingham; 8 Burnaby Centre for Mental Health and Addiction

## Abstract

**Aims:**

To identify the BOLD (blood oxygenation level dependent) correlates of bursts of beta frequency band electrophysiological activity, and to compare BOLD responses between healthy controls and patients with psychotic illness.

The post movement beta rebound (PMBR) is a transient increase in power in the beta frequency band (13-30 Hz), recorded with methods such as electroencephalography (EEG), following the completion of a movement. PMBR size is reduced in patients with schizophrenia and inversely correlated with severity of illness. PMBR size is inversely correlated with measures of schizotypy in non-clinical groups. Therefore, beta-band activity may reflect a fundamental neural process whose disruption plays an important role in the pathophysiology of schizophrenia. Recent work has found that changes in beta power reflect changes in the probability-of-occurrence of transient bursts of beta-frequency activity. Understanding the generators of beta bursts could help unravel the pathophysiology of psychotic illness and thus identify novel treatment targets.

**Method:**

EEG data were recorded simultaneously with BOLD data measured with 3T functional magnetic resonance imaging (fMRI), whilst participants performed an n-back working memory task. We included seventy-eight participants – 32 patients with schizophrenia, 16 with bipolar disorder and 30 healthy controls. Beta bursts were identified in the EEG data using a thresholding method and burst timings were used as markers in an event-related fMRI design convolved with a conventional haemodynamic response function. A region of interest analysis compared beta-event-related BOLD activity between patients and controls.

**Result:**

Beta bursts phasically activated brain regions implicated in coding task-relevant content (specifically, regions involved in the phonological representation of letter stimuli, as well as areas representing motor responses). Further, bursts were associated with suppression of tonically-active regions. In the EEG, PMBR was greater in controls than patients, and, in patients, PMBR size was positively correlated with Global Assessment of Functioning scores, and negatively correlated with persisting symptoms of disorganisation and performance on a digit symbol substition test. Despite this, patients showed greater, more extensive, burst-related BOLD activation than controls.

**Conclusion:**

Our findings are consistent with a recent model in which beta bursts serve to reactivate latently-maintained, task-relevant, sensorimotor information. The increased BOLD response associated with bursts in patients, despite reduced PMBR, could reflect inefficiency of burst-mediated cortical synchrony, or it may suggest that the sensorimotor information reactivated by beta bursts is less precisely specified in psychosis. We propose that dysfunction of the mechanisms by which beta bursts reactivate task-relevant content can manifest as disorganisation and working memory deficits, and may contribute to persisting symptoms and impairment in psychosis.

